# End-of-Life Discussions From the Perspective of Social Care and Healthcare Professionals in Palliative Care

**DOI:** 10.1177/00302228231185172

**Published:** 2023-06-21

**Authors:** Anne Kuusisto, Kaija Saranto, Päivi Korhonen, Elina Haavisto

**Affiliations:** 1 Department of Nursing Science, University of Turku Finland, Turku, Finland; 2 The Wellbeing Services County of Satakunta, Pori, Finland; 3 Satasairaala Central Hospital, Pori, Finland; 4 Department of Health and Social Management, 4344 University of Eastern Finland, Kuopio, Finland; 5 Department of General Practice, Turku University Hospital, University of Turku, Turku, Finland; 6 Health Sciences Unit of the Faculty of Social Sciences, 7840 Tampere University, Tampere, Finland; 7 Tampere University Hospital, Tampere, Finland

**Keywords:** end-of-life, death, health care professionals, living wills, palliative care, advance directives, advance care planning, interview, social work in health care, multicultural care context

## Abstract

This study describes the state of end-of-life discussions in Finland. A qualitative descriptive study with thematic interviews was conducted. Data were gathered from palliative care unit nurses, physicians and social workers. Inductive content analysis was used. According to interviewees (*n* = 33), the state of end-of-life discussion included three main categories. First, optimal end-of-life discussion time included early end-of-life discussion, end-of-life discussion at different phases of severe illness, and flexibility and challenges in scheduling end-of-life discussion. Second, end-of-life discussion initiators included both healthcare professionals and non-healthcare professionals. Third, social care and healthcare professionals’ experiences of end-of-life discussion consisted of the importance and challenge of end-of-life discussion, end-of-life communication skills development in multiprofessional care context, and end-of-life communication in multi-cultural care context. The results can be used to justify the need of a national strategy and systematic implementation on Advance Care Planning (ACP), considering the multiprofessional, multicultural and internationalizing operating environment.

## Introduction

For social care and healthcare professionals employed in palliative care units, it can be challenging to attain the treatment goals set for sensitive patients and families. Advance care planning (ACP) can help patients to understand their disease and prognosis and assist them in making decisions on end-of-life care (Goswami, 2023). ACP involves discussion of end of life, specification of relevant values and goals, and tailoring wishes with written records and medical orders (Institute of Medicine, 2015). The time remaining, psychosocial and physical considerations, and aspects of end-of-life care such as dignity constitute the contents of end-of-life discussion (Schüttengruber et al., 2022). An outline of systematic reviews shows that with the use of ACP, better end-of-life communication can be achieved (Jimenez et al., 2018). The need for early ACP, especially for patients with cancer diseases, has been identified and recommended (Ólafsdóttir et al., 2018; Kuusisto et al., 2020; Goswami, 2023). However, end-of-life discussions are rare, particularly in hospitals (Melin-Johansson et al., 2022), and they often occur too late (Lopez-Acevedo et al., 2013; Kuusisto et al., 2020; Mertens et al., 2021; Ikander et al., 2022) or not at all (Melin-Johansson et al., 2022), even during the end phase of cancer (Kuusisto et al., 2020).

End-of-life discussion is linked to culture and healthcare (Ha et al., 2021; Beck et al., 2023). Different cultures have different attitudes towards death in terms of how openly death is discussed (Travers & Taylor, 2016). Ethics (American Medical Association, 2018) and how ACP is guided by national laws (Act on the Status and Rights of Patients, 1992) and international recommendations (Institute of Medicine, 2015). While ACP is the shared responsibility of different stakeholders, no consensus exists on who should initiate ACP (Rietjens et al., 2021). Patients, family members (Travers & Taylor, 2016) and healthcare professionals each wait for the others to start the discussion (Institute of Medicine, 2015; Serey et al., 2022). A systematic review of systematic reviews shows that patients and family members hope that healthcare professionals will initiate the discussion (Hall et al., 2019). Most Norwegian adults wanted ACP to be made when a severe illness with restricted lifetime is diagnosed and wished that healthcare professionals would start it (Sævareid et al., 2021). Updating an ACP (Ikander et al., 2022; Serey et al., 2022) is needed when cancer progresses (Kuusisto et al., 2020). Several studies show that responsibility for starting the discussion rests with the physician who knows the patient best (Hall et al., 2019; Sævareid et al., 2021). A nurse (Mehta et al., 2018; Ólafsdóttir et al., 2018; Ikander et al., 2022; Sopcheck & Tappen, 2022), social worker (Stein et al., 2017; Ha et al., 2021) or a voluntary worker can also act as initiators of the discussion (Sellars et al., 2019).

Taken together, ACP is an established practice in many countries, such as the USA or the United Kingdom (Andreasen et al., 2019) whereas, for example, in Nordic countries, such as Norway (Sævareid et al., 2021), Iceland (Ólafsdóttir et al., 2018) or Sweden, ACP is not systematically implemented (Melin-Johansson et al., 2022; Beck et al., 2023). A new systematic review and metasynthesis of qualitative studies concluded that there is a need to compare ACP practices in different cultures to generate systematic implementation guidelines in different jurisdictions (Zhu et al., 2023). Thus, the aim of this study is to describe the state of end-of-life discussions in Finland. The research questions are:

1. What is the optimal time to discuss end-of-life issues?
2. Who should initiate end-of-life discussion?
3. What kind of experiences do healthcare professionals have of addressing end-of-life matters?

## Methods

### Study Design

The study made use of a qualitative descriptive study design. It is applicable particularly when a versatile and direct picture of the experiences of healthcare professionals’ practice is needed. (Neergaard et al., 2009; Bradshaw et al., 2017). The Consolidated Criteria for Reporting Qualitative Research (COREQ) were followed (Tong et al., 2007) (Supplementary File 1).

### Study Context

This study was carried out in three of the twenty hospital districts (as of 1 January 2023, known as Wellbeing Services Counties) in Finland. They offer public specialized medical care, including outpatient treatment, for about 40% of the Finnish population. In Finland, palliative care services are organized at basic (A), special (B) and demanding special level (B) according to the level of care required. In this study, the data were collected in special-level (B) units specializing in palliative care and end-of-life care. Their main task is palliative care and the staff is specially educated.

### Participants and Recruitment

Purposeful sampling was applied (Bradshaw et al., 2017; Gill, 2020). Palliative care is multiprofessional. For this reason, representatives from various professional groups who were assumed to have knowledge and experience of the phenomenon under study were selected as the target group in order to obtain a broad and versatile view (Gill, 2020). The voluntary interviewees were selected among clinical staff (licensed registered nurses, practical nurses, social workers and physicians) working in palliative care practice. A liaison person in each research unit acted as a contact person for the researchers and took care of selecting the interviewees. They also distributed research bulletins with information about the characteristics of the interviewees and permission forms.

### Data Collection

The interview method was utilized for data collection to obtain new and rich data according to qualitative descriptive study design requirements (Neergaard et al., 2009; Bradshaw et al., 2017). Focus group was the data collection method used with registered nurses and practical nurses. Physicians and social workers participated in one-on-one interviews because there was usually only one of them per unit. Two pair interviews were added for those who were unable to attend the focus group or one-on-one interviews. (Table 1)

**Table 1.** Demonstration of Data Gathering and the Study Interviewees (*n* = 33).

Interview Method	Professions	Code	Professions Represented (*n*)
One-on-one	Social worker	S1–S5	5
One-on-one	Physician	P1–P3^a^	3
Focus group	Practical nurse	FGPN1	5
Focus group	Registered nurse	FGN1	6
Focus group	Registered nurse	FGN2	4
Focus group	Registered nurse	FGN3	3
Focus group	Registered nurse	FGN4	3
Paired	Registered nurse	CIN1	2
Paired	Physician	CIP1	2

^a^“P3” = Telephone interview.

Thematic qualitative interviews were carried out with vocational group-specific face-to-face sessions in the natural setting, i.e., the workplace; one was a telephone interview and one was conducted in a more personal location suggested by the professional without interference from other people. The data were compiled from May to November 2019 in six palliative care units in three hospital districts. The interviewees gave permission to record the interviews which lasted on average 58 minutes. For the interviews, a themed interview guide was created, pilot-tested and followed (Table 2). In addition, the interviewees filled out a short background information form.

**Table 2.** Themes as the Framework for End-of-Life Discussions.

Themes
Optimal time to discuss end-of-life issues (e.g., phase of illness, healthy)
Initiators of end-of-life discussions (e.g., physician, nurses, social worker, patient)
Conveying patient information - subjects relevant to end-of-life discussions were analyzed
Development areas of palliative care - development areas concerning end-of-life discussions were analyzed

### Data Analysis

The data concerning optimal time to discuss end-of-life issues (RQ1) and the data concerning experiences of addressing end-of-life matters (RQ3) were analyzed using inductive content analysis. It is suitable for use when there are insufficient previous research data on the studied phenomenon (Bradshaw et al., 2017.) The data on persons initiating end-of-life discussions were analyzed separately by professional and non-professional group (RQ2). Interviewees were assigned numbers to protect their anonymity. In addition, the numbering made it possible to trace the original answers and return to them during the analysis process. A meaningful statement or a set of facts was chosen as the unit of analysis. After that, simplified expressions were formed from meaningful statements or entities, and those with similar content were classified into subcategories and further into categories and main categories (Tables 3–5). The categories were named according to their content.

**Table 3.** Optimal End-of-Life Discussion Time.

Main Category	Category	Sub-category
Optimal end-of-life discussion time	Early end-of-life discussion	Discussion when person is healthy
		Discussion at the time when the patient is capable of presenting end-of-life wishes
	End-of-life discussion at different phases of a severe illness	Discussion in the early phase of a severe illness
		Discussion during the worsening phase of a severe illness
		Discussion during the final phase of a severe illness
	Flexibility and challenges in scheduling of end-of-life discussion	Individual discussion timetable
		Difficulty of defining an optimal time for discussion

**Table 4.** End-of-Life Discussion Initiators.

Main Category	Category	Sub-category
End-of-life discussion initiators	Healthcare professional as end-of-life discussion initiator	Physician as end-of-life discussion initiator
		Nurse as end-of-life discussion initiator
		Physician and nurse jointly responsible as end-of-life discussion initiators
	Non -healthcare professional as end-of-life discussion initiator	Patient or family member as end-of-life discussion initiator
		Voluntary worker or hospital pastor as end-of-life discussion initiator

**Table 5.** Social Care and Healthcare Professionals’ Experiences of End-of-Life Discussion.

Main Category	Category	Sub-category
Social care and healthcare professionals’ experiences of end-of-life discussion	Importance and challenge of end-of-life discussion	Importance of providing truthful information
		Challenges of talking about end-of-life
	End-of-life communication skills development in multiprofessional care context	Importance of developing end-of-life interaction skills between social care and healthcare professionals
		Educational interventions for developing end-of-life discussion skills
	End-of-life communication in multicultural care context	End-of-life communication challenges between different cultures
		End-of-life communication challenges related to the lack of a common language

### Ethical Aspects

The researchers followed the ethical regulations of the Declaration of Helsinki including research permits and confidentiality throughout the research process (World Medical Association, 2023). Before starting the research, ethical approval was obtained from the ethics committee (15/2019). The interviewees were informed about the study in advance. Oral and written informed consent were taken at the time of interview attendance. All the interviews were audiotaped with permission and transcribed verbatim. The interviewees were allowed to withdraw from the study any time.

## Results

The age of the participants (*n* = 33) varied from 19 years to 62 years (mean 46 years). They had been working in healthcare for an average of 17 years (range from less than 2 years to 37 years) and in palliative care for an average of 6 years (range from less than 1 year to 19 years). More than half of the participants (67%) had participated in additional palliative education.

### Optimal End-of-Life Discussion Time

The optimal end-of-life discussion time as assessed by professionals working in the palliative unit included three categories (Table 3).

### Early End-of-Life Discussion

Early end-of-life discussion meant discussion when the person is healthy and on the other hand, discussion at a time when the patient is capable of expressing end-of-life wishes (Table 3).

*Discussion when the person is healthy* implied that ideally, end-of-life care matters should be discussed before getting ill, i.e., when individuals are not yet patients, which means now. The interviewees considered that there are things that can be anticipated. For example, everyone can make a living will while healthy. In a living will, a person can define, for example, whether they want to be on a ventilator for a long time or die if there is no hope of recovery. Professionals said that these issues can be discussed with adults, whereas persons under 18 years were assessed to be too young for such discussion.

*The ideal time would be when the patient is still, like, in their senses and quite healthy, which means “now”. (FGN4)*

*But in the case of a healthy person, a living will can now be made by anyone. (S5)*

*Discussion at a time when the patient is capable of presenting end-of-life wishes* meant that end-of-life matters should be discussed when patients are able to cooperate, i.e., able to speak and express their own wishes. If the disease progresses quickly, patients’ own wishes may no longer be ascertained. Professionals said that especially if a patient is in the hospital, end-of-life matters should be discussed fairly soon. They had strongly encouraged patients to prepare living wills in advance, while they still could. In the hospital, preparing a living will was assessed to be easy. Professionals had advised patients to think about what kind of end-of-life care they wished to have without having to make decisions. Professionals revealed that in hospital, they had cared for many patients who had never accepted the deterioration of their situation or shared their end-of-life wishes. In addition, they said that if patients had not expressed their wishes, their family members were left guessing. In that case, they did not necessarily know what the patient had actually wanted. Thus, professionals saw it as very difficult for families to make big decisions on behalf of their loved ones if they were not aware of their wishes.

*Of course, they should be discussed when the patient is still able to speak and able to express their wishes and thoughts. (CIN1)*

*As for myself, I’ve held a clinic for people with memory problems, so I want to bring it up and say that you should make it as long as... (CIP1)*

*…so they should really think about it when they are still all right, so they consider what they want (FGN2)*

### End-of-life Discussion at Different Phases of a Severe Illness

Discussion at different phases of a severe illness meant discussion in the early phase, discussion during the worsening phase, and discussion during the final phase of a severe illness (Table 3).

*Discussion in the early phase of a severe illness* referred to the optimal time for discussion of end-of-life issues in connection with the diagnosis of a severe illness. Professionals said that end-of-life issues should discussed at the latest when the patient is diagnosed with a fatal illness. It was seen as particularly important if a patient or a family member brought up end of life and wished to do so. In practice, the topic of death could be approached through leading questions. For example, by inquiring about general issues, such as whether the patient had talked to their children about forthcoming death and funeral. Despite their own doubts, professionals pointed out that sometimes it can be quite easy to bring up death with a sick person who has recently been confirmed to have a fatal illness and is confused about the situation.

*They should be, at least in most cases, when diagnosed and when the serious, fatal…(CIP1)*

*…so right at the time when the illness that will lead to death has been diagnosed (S3)*

*…if the patient has been quite recently diagnosed and knows, so they are still in a state of shock as it were, that’s when it’s sometimes pretty easy to bring it up and ask what they think about death (FGN2)*

*Discussion during the worsening phase of a severe illness* meant that end of life should be discussed at life’s turning points. For example, when symptoms of a disease increase and patient’s functional ability has weakened so much that there is a need to assess the need for regular outside help with daily activities to cope at home. One of the interviewees had worked in home care and suggested that a living will form should be completed routinely for everyone when assessing the need for home care. If a living will existed it would be easy to return to it later. In the case of cancer, it was felt to be easier to identify the time of end-of-life conversation than in other diseases leading to death. The discussion with the patient with cancer should take place when life-extending treatments were underway. According to the professionals, if patients were suffering from, e.g., heart failure, the right time to raise end-of-life issues was when there were several exacerbations of the disease in a year. The professionals said that issues and wishes related to death could be discussed even if the patient was not yet in the end-of-life phase. Early contact with palliative care was also seen to be associated with a longer end-of-life phase.

*And at the stage when the symptoms start to appear, and the patient’s functional ability is definitely deteriorating. (P1)*

*…if you need outside help, well in my opinion that’s a sign that you should think about things like that. (CIP1)*

*Discussion during the final phase of a severe illness* meant that according to the interviewees, in general, patients in need of palliative care were identified too late in the service system. That is why end-of-life issues were brought up too late. The professionals said that discussions should clearly take place at an earlier phase of disease than is currently the case. In any case, it should not under any circumstances happen during the end-of-life phase. End-of-life discussion was seen as appropriate when a person could no longer cope at home, had to move to a long-term care facility, or had to be permanently hospitalized. According to the interviewees, matters concerning end of life should be discussed with the family members at the latest when the patient moves to long-term care, enhanced assisted living or a palliative care unit. In the view of the professionals, end-of-life discussion may even be easier in the palliative ward. That is because the patients are aware that they are coming to the palliative care unit in the final phases of their disease. On the other hand, depending on the patient, moving to a palliative care unit can be a big shock. One interviewee said that she/he would like to have the discussion when there is no longer any curative treatment available. According to the professionals, matters concerning end of life should be discussed at the latest when a palliative treatment line decision has been made or before sedation. On the other hand, at what point a palliative treatment plan should be made was seen as a different matter.

At times, patients had come to the palliative unit from the emergency department (ED), where they had been told that the situation was very bad. They had been moved from ED without any mention of death and the very high likelihood that they would die. In practice, they had been moved from ED to a palliative unit where they eventually died; at that point, there were questions as to why this happened. It was considered important that patients in the palliative phase should be identified at an early phase in the service system and a referral to palliative care be made. The fact that physicians did not recognize patients in the palliative phase was identified as a challenge. According to the interviewees, physicians’ training emphasizes curing diseases and the aim is to help the patient to get better. That is why palliative specialist physicians were quite often only consulted in the end-of-life phase, even though these patients would have benefited from palliative consultation at an earlier stage. The interviewees thought that end-of-life issues were quite rarely discussed at a specialist clinic where the focus was on the treatment of the patient’s underlying disease.

*…that if they can no longer manage at home and then go to a place of care, I think that is the last chance to discuss the situation with the next of kin, when they are moved to long-term care or an assisted living facility... or to a palliative care unit. (S5)*

*… in the ER they have said something like ‘the situation is really bad’, and the patient is sent here, but they have never talked about dying there, the very high likelihood of death, and then in practice, they come in here and die, and then everyone wonders why it happened. (FGN2)*

*…at a clearly earlier stage than is now the case. So absolutely not in the palliative care phase… (P2)*

### Flexibility in Scheduling End-of-Life Discussion

Flexibility and challenges in scheduling the discussion included individual discussion timetable and difficulty defining the optimal time for discussion (Table 3).

*Individual discussion timetable* meant that the optimal scheduling of talking about the end of life depended on the patients and their condition. Professionals saw these discussions as necessary. They stated that the time when the discussion regarding the end-of-life issues was held did not matter as long as it had taken place. Moreover, they said that the end-of-life decision should always include a discussion. In practice, end-of-life issues were being arranged all the time. Even if the end-of-life decision was made just before the patient’s death, there might be changes to it because it was dynamic and the patient’s wishes might be complemented and change slightly over time. The interviewees pointed out that not all patients wanted to talk about end-of-life matters at all, so they were not always discussed. In that case, the professionals respected the patient’s dignity. They recorded that the patient did not want to discuss it and they did not know how to define their wishes at that moment.

*It really depends on the case. And they can be complemented and change slightly along the way. (FGN3)*

*...that whatever the phase at which it’s made, there’s always that discussion, but of course it’s never, even if made towards the end, before the person dies – there may still be changes, it’s dynamic, as it were. (FGN1)*

*…some don’t want to talk about them at all. (FGN4)*

*Difficulty of defining an optimal time for the discussion* meant that professionals were required to be situationally aware when choosing a suitable time for the discussion. The optimal timetable was described as particularly challenging, particularly if the patient was being treated in the palliative ward and if end-of-life issues had not yet been discussed with the patient and family members. Professionals said that end-of-life matters should be inquired subtly. They said that they waited for the right moment. For example, if a patient felt anxious, professionals could ask general questions about their state of health, whether everything was fine, or whether the patient wanted to talk about something. However, not all the interviewees were able to express an opinion on the optimal time for the end-of-life discussion.

*…the patient is on the ward and the next of kin, the next of kin – if they are not yet there at that time you need to be sensitive and consider at what point these things should be discussed (FGN4)*

*At least personally, I wait for the right moment. It could be asking about things in a roundabout way, like how are you feeling today, and if you sort of see that the person is distressed, asking if everything is okay and is there something you want to talk about. So you are fishing for information, like (FGN2)*

### End-of-Life Discussion Initiators

End-of-life discussion initiators included two categories (Table 4).

### Healthcare Professional as End-of-Life Discussion Initiator

*Physician as end-of-life discussion initiator* meant that discussion was seen as the physician’s responsibility, particularly if it relates to medical issues such as treatment lines or matters concerning the end-of-life care phase. A physician was also appointed to discuss medical decisions (e.g., DNR). Physicians were also judged to be the most natural choice to address end-of-life matters because they can evaluate the patient’s prognosis and different treatment options. The physician with overall responsibility for a patient’s care was considered to have the responsibility, particularly if there had been no discussion before. In such a case, the physician responsible for the treatment can refer the patient to follow-up by a palliative physician. The interviewees pointed out that the physicians decide the optimal time for the discussion from their own perspective.

*If it has to do with an illness-related decision, well in that case I guess it has to be made by a physician. (CIP1)*

*…a task for physicians when it comes to medical DNR decisions and such. (CIN1)*

*…thoughts about that we are now in the palliative care phase etc. etc., they are all matters for the physician. And things that the physician evaluates. (FGPN1)*

*In general, maybe it's most natural for the physician to bring it up, because the physician knows about the prognosis and the possibilities with different treatment options. (S3)*

*Nurse as end-of-life discussion initiator* meant that a nurse can independently talk about the end-of-life matters or notify the physician that there might be a need to discuss these issues. The interviewees pointed out that the most natural thing is not to leave everything to the physician, as nurses are constantly closest to the patients and on site 24/7. The interviewees pointed out that some patients can talk about end-of-life subjects to the physician as well, but in practice, they talk more to nurses. The nurse may remind the patient that if they do not desire to talk about the end of life at the moment, they will have the opportunity to talk about it at a time appropriate to them, and they do not have to wait for the physician to arrive the next day. All nurses and, on the other hand, the nurse with whom the patient has a confidential relationship, have a responsibility to initiate the discussion. If a patient had a good relationship with the nurse, the nurse will be able to prepare the matter. The professionals reported that it may be easier for a nurse (than a physician) to bring up end-of-life matters if the patient is withdrawn or anxious. The interviewees revealed that a nurse should refer to end of life in concrete terms. The task of nursing professionals was, for example, to talk about nursing issues, i.e., how the patient wants to be cared for.

*So, the nurse can bring it up, but the nurse can also give a nudge to the physician, that maybe this should be brought up. (P2)*

*But on the other hand, we nurses are there 24/7 so I think it’s quite natural that not everything should be left to the physician. (FGN3)*

*Some may talk to a physician, but well – I’d say that patients talk more to nurses. (FGPN1)*

*…after all, nurses are like closest at hand and we are here all the time, so you can always remind them that if you don’t want to talk about these things right now, and then when you feel like it, you have a possibility to do it – you don’t have to wait until the next day for the doctor to come or… (FGN4)*

*Physician and nurse jointly responsible as end-of-life discussion initiators* meant that discussion was felt to be part of the work duties of physicians and nurses. Professionals said that whose responsibility it was depended on the patient’s situation. The person could be the patient’s own physician, sometimes a nurse, but less often a practical nurse. As for a living will that patients can make themselves, any healthcare professional could bring it up. Professionals said that when a patient arrives in the palliative ward both nurses and physicians talk with them about end-of-life matters. These topics were also discussed in care meetings where many issues might come up.

*It is a task for all of us. (FGPN1)*

*But if we are talking about a living will which the patient can make personally, that can be brought up by anyone working in health care. (CIP1)*

### Non Healthcare Professional as End-of-Life Discussion Initiator

*Patient or family member as end-of-life discussion initiator* meant that patients and in practice, according to the interviewees, even more frequently, family members, were the ones who initiated the discussion and made the first contact. This indicated that matters concerning end of life were discussed in a patient-oriented manner, i.e., if the patient or family member wished to do so. The professionals did not actively go out to offer to initiate discussion; instead, they proceeded according to the situation. For example, a social worker could initially explain to the patient or family member in detail what kind of issues the social worker could check and help with. In this case, when the patient was in the end-of-life phase, a very sad family member was told about the possibility of having a psychosocial support session.

*There are some who bring up their own wish like right away. (P2)*

*Well, I’ve chosen the approach that it starts with the patient or their next of kin, so that is where we begin. (S1)*

*Voluntary worker or hospital pastor as end-of-life discussion initiator* meant human resources whose role was often perceived as an important asset alongside healthcare professionals in a palliative unit. Occasionally, voluntary workers were the only ones who had succeeded in initiating the discussion on end-of-life matters, such as patients’ wishes regarding their treatment or funeral. Sometimes, voluntary workers had been able to initiate discussion on end-of-life questions with the patient. This was a relief for the family members, who were then also able to initiate discussion on the matter. In the palliative unit, there were also hospital pastor services available. Professionals could ask if the patient wanted to talk to a hospital chaplain, for example.

*…a volunteer had managed to initiate the discussion and it continued from there, and the wife said it was such a huge weight off her shoulders that the matter had been brought up, and now she would be able to bring it up herself (FGN4)*

*…we have hospital pastor services available, so you can also ask if they wish to talk to a chaplain. (P3)*

### Social Care and Healthcare Professionals’ Experiences of End-of-Life Discussion

Healthcare professionals' experiences of end-of-life discussion included three categories (Table 5).

### Importance and Challenge of End-of-Life Discussion

*Importance of providing truthful information* meant that in the opinion of professionals, speaking honestly was seen to be the best method. According to them, discussion was important in order to know how things are progressing. It was considered important that physicians inform patients honestly about their situation. On the other hand, it was said that when talking about difficult things, it would be good to prepare the patient and ask for permission to talk. That was pointed out because according to professionals, Finnish patients generally wish to be told honestly what their situation is. It is a characteristic feature of Finnish culture to call things by their real names. The interviewees said that, for example, when they talk about death, they should talk about death and not about something vague. At times, talking about death was considered a form of therapy. It is not scaremongering; it is a fact that is plain for everyone to see. According to the interviewees, documentation made it visible that the issues in question had been discussed and considered. Unless healthcare professionals bring up end-of-life issues, the patient thinks that they are taboos that cannot be talked about.

*But of course, it means that the doctor must be honest when informing the patient about the situation. (S2)*

*…things should be discussed using their real names, so that when you talk about death, you talk about death, and not about something vague. (P3)*

*Challenges of talking about end-of-life* concerned both social care and healthcare professionals and patients who described awkwardness of talking about death. Professionals generally considered talking about end of life as foreign, something they did not always have the courage to do. Professionals said that not even one in ten was able to initiate end-of-life discussions. Indeed, those who had worked in palliative care for a long time said that it was sometimes very demanding. Despite the fact that talking about death was considered somewhat natural in the palliative care unit it can be quite difficult to discuss issues, arrangements and wishes related to death with patients, unless patients themselves bring them up at some point. According to the interviewees, people have a natural tendency to think that “yes, I can still get through this.” In these situations, professionals felt it was cruel to bring up end-of-life matters, even though they thought that it would be reasonable. Potentially more challenging were situations where patients go to a non-palliative unit for some other reason (than death) and are no longer able to go home because the fatal disease has progressed.

Talking about end of life took time and required listening. One physician said that the patient may not respond immediately, but possibly later, e.g., the next day. Professionals revealed that when they had brought up the fact that the disease would end in natural death, quite a large proportion of the patients had already understood it and were aware of it. However, those patients had not dared to bring up death because they had been told that the objective of suppressive treatment was to prevent the progression of the disease. Moreover, there were still situations where even physicians had not dared to use the concept of death. Professionals said that situations in which patients wanted to know their prognosis and how much time they had left were very challenging. The interviewees said that at least once a week there was a patient who wanted to know the exact date and remaining time. However, professionals were not able to give an answer about remaining time, because the disease progressed individually in different patients.

*In a sense, these are things that we should be able to discuss quite openly in the ward, but it’s not easy. And it’s, like, not easy to open them either. (S4)*

*…not even doctors have always dared to use the word “death.” (FGN2)*

### End-of-Life Communication Skills Development in Multiprofessional Care Context

*Importance of developing end-of-life interaction skills between social care and healthcare professionals* touched on both oral and written communication. In the case of healthcare professionals, it was seen as important to move on from old-fashioned ways of operating, e.g., the idea that nurses cannot independently consult palliative care physicians. According to physicians, nurses should not be afraid to tell the physician if they have recognized the need for palliative consultation. Similarly, physicians should listen more to nurses and read their records. Nurses see patients daily, around the clock. From a social worker’s point of view, they did not meet all the patients coming to the palliative care unit, but they had usually talked with new patients at the hospital on an as-needed basis. After patient discharge, social workers were usually not contacted about anything, unless there was something immediate, urgent. According to social workers, they should be included in multi-professional discussions, especially at the stage when a decision is made for the patient to switch to a symptomatic treatment line, i.e., at the point when the patient’s illness progresses, their condition deteriorates, and no response can be achieved with medication.

*… so that at least, nurses would not be afraid of telling the doctor that in a situation like this, it might be a good idea to consult someone. (P1)*

*So the nursing staff and the doctor should, like, observe, that now, this patient ... that now we have to make a decision to move on to a symptomatic treatment line, and that is the time when a social worker should be involved as well. But contacts like that occur much more rarely. (S1)*

*Educational interventions for developing end-of-life discussion skills* included different ways in which interaction between professionals and patients can be achieved and developed. Basically, work experience was estimated to help develop end-of-life discussion skills. The interviewees revealed that at the beginning of their career, they did not always know how to discuss end-of-life problems. They also considered it important to include end-of-life discussion training in nursing education. It could be the kind of education that has for a long time been included in various phases of medical education. Instead of only lectures, teaching analysis of practical end-of-life patient cases and especially, mutual training of professionals assisted by an interaction professional in small groups, were considered useful methods in continuing education of end-of-life discussion skills. Personal training with learning by doing was evaluated as a useful way to learn end-of-life interaction skills than patient cases presented by actors. On the other hand, not everyone was considered to ever learn end-of-life communication skills. All in all, interpersonal end-of-life communication was thought to come more naturally to some people than others.

*…at the beginning, nobody really knew how these things should be brought up in discussion. (FGN3)*

*It’s, like, done in small groups, it’s not something that you hear during lectures. (P2)*

### End-of-Life Communication in Multicultural Care Context

*End-of-life communication challenges between different cultures* were connected to ethical issues, such as patient’s rights and self-determination. Getting and giving information was seen as a patient’s right. If patients said they did not want to receive information, they were seen as having the right to refuse it. If the patient’s culture was such that one should not talk about death, then it was considered to be the patient’s right. There were a few patients from multicultural families, to whom professionals should not talk about death, for example; in such cases, the nurses were in a challenging situation. Sometimes a family member of a patient from a multicultural family insisted that the patient should not be told that their situation is bad. According to the nursing staff, the patient had the right to refuse and not follow the instructions given by them, even if the nursing staff found it difficult. The nurses described it as the most difficult when they wanted to help the patient by giving them all possible information. Nurses have adopted the principle that if a patient refuses to take the information offered or does not act in accordance with it, it is their right. In those cases, they thought about how to act.

*And they have a right to refuse our instructions, whatever information, they have the right not to follow it, even though we find it difficult. (FGN1)*

*The patients should not be told that they are in poor health, the next of kin forbid it, and how to deal with that. (FGN1)*

*End-of-life communication challenges related to the lack of a common language* meant that the unit also treated patients from other countries than Finland. Sometimes it was very difficult to have end-of-life discussions because the nurses, patients and families did not always have a common native language between them. A common language (mother tongue) was considered very important. If the patient’s Finnish language skills were very weak and there was no common language, the professionals could not be sure what the patient understood and wanted to express. In a serious situation, without a common native language, it can be a challenge for the patient to make herself or himself understood, due to cultural differences, for example. According to the professionals, even the use of an interpreter did not always solve the challenges of end-of-life discussions. Sometimes there were also communication challenges between the interpreter, patient and nursing staff. The interpreter may have refused to convey information to the patient or expressed the matter in a different form than the nurses, because the information was not in accordance with the culture in question. The interpreter may also have left in the middle of the meeting or may not have turned up at all.

*…it is always a challenge in a serious situation like this to make yourself understood by the patient, and then there are all kinds of cultural differences and things like that. (CIN1)*

*Or when we are using an interpreter who refuses to pass on the information as we give it because in that culture, it is not done, or who takes off during the meeting or does not show up at all. (FGN1)*

## Discussion

In this study, social care and healthcare professionals express that early ACP is preferable. They hope that end-of-life discussion would be done as early as possible during the disease, or even before getting ill in the form of a living will. Here, primary healthcare, and more broadly, society as a whole, have a big task to expand their use, especially in Europe (Serey et al., 2022). An early end-of-life discussion at a time when, for example, patients with cancer are not too sick (Ólafsdóttir et al., 2018), can help them reflect on a living will (Serey et al., 2022) as well as provide emotional (Goswami, 2023) and discharge (Mertens et al., 2021) support. Early discussions have a connection with the quality of life, such as fewer acute treatments like chemotherapy or intensive care periods at the end-of-life stage (Lopez-Acevedo et al., 2013). On the other hand, physicians’ late end-of-life discussions have hindered multi-professional teamwork and patients’ participation in shared decision-making (Mertens et al., 2021).

Contrary to international guidelines (Institute of Medicine, 2015) and compared to the previous American or Korean studies (Stein et al., 2017; Ha et al., 2021), in this study there were no mentions of social workers as initiators of end-of-life discussions. Instead, social workers directed patients and family members to talk to a physician. This may arise from medicine-oriented specialized (palliative) care and the fact that the role of social workers in Finnish palliative care may not be clear or is not used enough. According to this and many other studies (e.g., Hall et al., 2019; Sævareid et al., 2021), all professionals in this study seem to think that that physician-initiated ACP is more effective. On the other hand, at the same time, the interviewees admit that it is often easier for the patient to talk to a nurse who is present in the ward around the clock. Recently, Rietjens et al. (2021) showed that unclear responsibilities for ACP initiation and expectations that a physician should start ACP have been identified as barriers to ACP. Another study by Sopcheck and Tappen (2022) showed that nurses’ subtle observations of a patient’s deterioration can initiate discussion about the end of life.

In this study, as expected based on previous studies (Travers & Taylor, 2016; Hall et al., 2019), psychological barriers impeded discussion of ACP, but unexpectedly, end-of-life communication in the multicultural care context with language barriers is a surprising finding which was not predicted or hypothesized beforehand. Reflections and questions related to death are challenging even for experienced palliative care professionals. The interviewees stated that Finnish patients generally appreciate honest discussion about their situation. Moreover, talking about things other than death with the patient can sometimes lead to a conversation about death. Conversation with patients is important, because it makes it easier for them to discuss issues related to death with their family members as well. In addition, according to previous studies, insufficient education and training of healthcare professionals, especially nurses (Ikander et al., 2022), has hindered the discussion of end-of-life issues (Travers & Taylor, 2016). Both physicians and nurses in this study described problems caused by the lack of a common language regarding end-of-life discussions with foreigners and patients from a foreign culture. Even the help received from an interpreter did not always solve the problems in question. This is noteworthy because, according to ethical principles (American Medical Association, 2018) and legislation (Act on the Status and Rights of Patients, 1992), all people have an equal right to receive palliative care according to their needs. In terms of the quality, continuity and safety of treatment and care, it is important that the interaction between the staff, the patient and their families goes smoothly. We present some proposals of how to implement ACP despite the difficulties. In line with this study, the need for practical and practice-based training has also been expressed in a previous study (Travers & Taylor, 2016) to provide readiness for initiating discussion about end-of-life issues (Mehta et al., 2018). The development of multicultural competences should also be taken into consideration in palliative care in a globalizing world. Patient’s language and culture should be taken into account in palliative care. Moreover, a national strategy on ACP is needed to support systematic implementation of ACP in the new integrated Finnish social care and healthcare practice, taking into account the multiprofessional, multicultural and internationalizing operating environment.

### Strengths and Limitations

Qualitative description is a suitable method for research questions “who” and “what”, which were used in this study (Neergaard et al., 2009). An appropriate and purposeful qualitative sample of knowledgeable representatives of various social care and healthcare professional groups strengthened the accuracy of the data collection, which ended when the data started repeating itself. On the other hand, one limitation may be the fact that there were four interviewers, although the instructions were reviewed jointly by them. Another limitation may be that one researcher (first writer) coded the data and performed the analysis; however, the analysis was reviewed by the research team concerning the categories. Moreover, citations of the interviewees’ authentic responses strengthen the reliability of the analysis (Bradshaw et al., 2017).

## Conclusions

This study shows end-of-life discussion practices in the Nordic context in Finland. Early end-of-life discussion with psychosocial support, or even before getting sick in the form of a living will, are important parts of successful palliative care. All professionals also seem to think that initiating end-of-life discussion is the physician’s responsibility, but at the same time, they admit that it is often easier for the patient to talk to a nurse. In the future, there is a great need to study and develop effective practical educational interventions for discussing end-of-life matters. The results of this study can be used to justify the need of a national strategy and the systematic implementation on Advance Care Planning (ACP), taking into account the multiprofessional, multicultural and internationalizing operating environment.

## Supplemental Material

Supplemental Material - End-of-Life Discussions From the Perspective of Social Care and Healthcare Professionals in Palliative Care Supplemental Material for End-of-Life Discussions From the Perspective of Social Care and Healthcare Professionals in Palliative Care by Anne Kuusisto, Kaija Saranto, Päivi Korhonen, and Elina Haavisto in OMEGA - Journal of Death and Dying.
